# Found: The Elusive ANTAR Transcription Antiterminator

**DOI:** 10.1371/journal.pgen.1002773

**Published:** 2012-06-07

**Authors:** Valley Stewart, Herman van Tilbeurgh

**Affiliations:** 1 Department of Microbiology, University of California Davis, Davis, California, United States of America; 2 IBBMC-CNRS UMR8619, Université Paris-Sud, Orsay, France; Agency for Science, Technology, and Research, Singapore

Regulated transcription termination provides an efficient and responsive means to control gene expression [1]. Intrinsic terminators, which consist of an RNA stem-loop followed by a poly-U tract, catalyze termination by disrupting the RNA polymerase elongation complex [2]. In antitermination, an antiterminator stem-loop is mutually exclusive with the terminator stem-loop [1], [3]. In different contexts, formation of the antiterminator stem-loop is governed by a translating ribosome [1], a ligand-binding riboswitch [4], [5], or a signal-responsive RNA–binding protein [3]. This latter mechanism is illuminated by Ramesh et al. [6], who studied antitermination by a broadly distributed class of signal-responsive RNA-binding proteins containing the ANTAR domain.

## AmiR and NasR Define the ANTAR Regulators

Studies conducted in the 1990s characterized two related antitermination proteins: AmiR, which regulates aliphatic amide catabolism in *Pseudomonas aeruginosa* (*amiECBRS* operon) [7], [8], and NasR, which regulates nitrate assimilation in *Klebsiella oxytoca* (*nasFEDCBA* operon) [9], [10]. RNA binding occurs through the carboxyl-terminal ANTAR domain (AmiR and NasR transcription antitermination regulator) [11]. AmiR and NasR RNA binding responds to signal input mediated by the amide-binding AmiC protein [12], [13] and the nitrate-binding NIT domain [14], [15], respectively.

**Figure 1:**
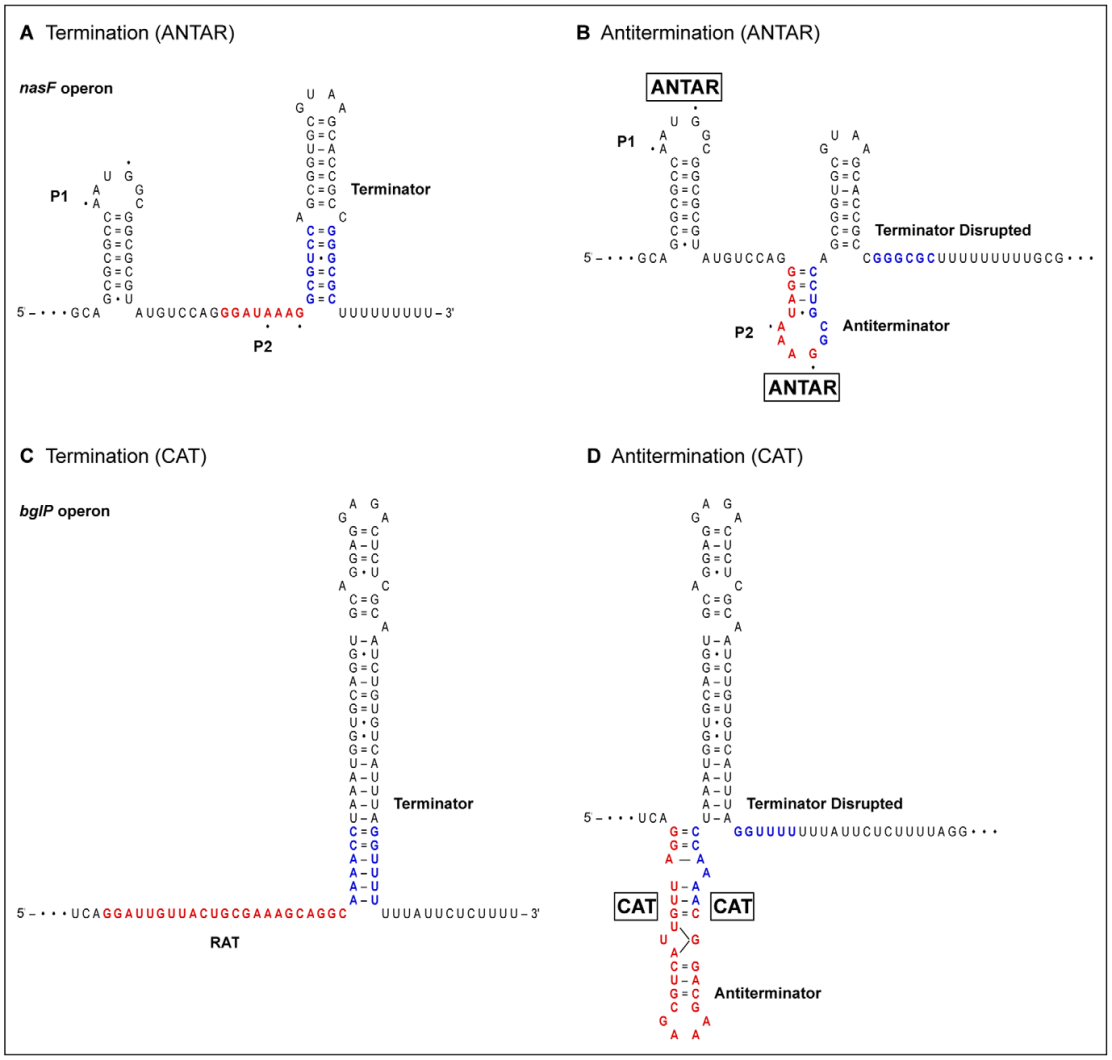
RNA-binding proteins stabilize antiterminator structures. Alternative stem-loop structures in transcribed leader regions from representative operons are shown. Terminator stem regions that participate in alternative structures are in blue, whereas antiterminator regions are in red. (A) NasR-responsive *nasF* operon leader in termination conformation. Dots indicate the critical residues A1 and G4 in the P1 and P2 loops. (B) Each ANTAR monomer is hypothesized to bind one of the loops, P1 and P2. Stabilizing the P2 antiterminator structure permits transcription readthrough [6] by shortening the terminator stem and separating it from the poly-U tract [2]. (C) LicT-responsive *bglP* operon leader in termination conformation. RAT is the ribonucleic antiterminator. (D) Each CAT (co-antiterminator) monomer binds to the antiterminator stem. Unusual base-pairing within the antiterminator stem is depicted schematically [25]. Stablilizing the antiterminator structure permits transcription readthrough [22] by shortening the terminator stem and separating it from the poly-U tract.

AmiR and NasR target the transcribed leader RNA upstream of the *amiE* and *nasF* operons, respectively [12], [14]. Both leaders encode two obvious stem-loop secondary structures: the distal intrinsic terminator (including a poly-U tract) [2], and P1, a proximal structure essential for antitermination (Figure 1A). Further analysis of the *nasF* operon leader identified three subelements essential for NasR binding and antitermination: the P1 stem; residues A1 and G4 in the P1 loop; and part of the linker region that connects the P1 and terminator stem-loop structures [16]. However, the RNA secondary structure(s) formed during antitermination remained a mystery.

Although different ANTAR proteins use a variety of signal input domains, at least half contain a two-component signal transduction receiver domain [6], [11]. Receiver function is governed through phosphorylation and dephosphorylation by a cognate signal-responsive sensor kinase [17]. For example, the ethanolamine-responsive EutW sensor kinase controls the EutV response regulator, which mediates antitermination control of ethanolamine catabolism (*eut* operons) in *Enterococcus faecalis* [18]–[20]. Other ANTAR proteins, including AmiR, contain a non-phosphorylated pseudo-receiver domain [13], [17].

## New Progress with EutV

Ramesh et al. [6] now report a substantial advance toward understanding ANTAR-mediated transcription antitermination. The *eut* gene cluster contains intrinsic terminators upstream of four genes (*eutP*, *eutG*, *eutS*, and *eutA*), facilitating the identification of shared sequence features. Each of these leaders contains a terminator along with two upstream stem-loop structures, P1 and P2 [6] (Figure 1B). The *eutG* leader P2 structure was noted independently by Baker and Perego [20]. Similar structures are present in the *amiE* and *nasF* leaders. Strikingly, both the P1 and P2 loops contain the critical residues A1 and G4, identified as essential for NasR-mediated antitermination [16]. Moreover, the distal stem of P2 overlaps part of the terminator proximal stem [6], [20], and thereby forms an antiterminator structure (Figure 1B). This suggests a general model for ANTAR-mediated transcription termination, in which the dimeric ANTAR protein binds simultaneously to structures P1 and P2, stabilizing the P2 antiterminator to enable transcription readthrough. (Indeed, restriction sites introduced into the *nasF* operon leader [16] destroy the P2 structure, explaining the resultant uninducible phenotype.)

To test this model, Ramesh et al. first constructed a variety of site-specific alterations in the *eutP* leader, and confirmed the subelements important for EutV binding and antitermination: the P1 and P2 structures; their relative spacing; and the P1 and P2 loop residues A1 and G4 [6]. Ramesh et al. then evaluated the EutV oligomeric states, finding that the wild-type protein required phosphorylation to dimerize. Truncated proteins lacking the receiver domain were isolated as dimers, although the most efficient RNA binding was observed with truncated protein that retains the central coiled-coil. This suggests that the coiled-coil enforces proper spatial orientation between the two ANTAR monomers. Presumably, each ANTAR monomer binds one of the two structures, P1 or P2.

Finally, Ramesh et al. conducted bioinformatic analyses to identify ANTAR target leaders in bacterial genomes. Examples were found in a broad range of species from the Actinobacteria, Bacteroidetes, Firmicutes, and Proteobacteria. Some of the leaders are adjacent to genes of unknown or uncertain function, whereas many others are adjacent to genes involved in inorganic nitrogen acquisition (ammonium, nitrate, or dinitrogen) or assimilation (glutamine synthetase and associated regulators). Many ethanolamine utilization clusters also were identified.

Together, these results provide concrete hypotheses for the structure and function of both ANTAR domain proteins and their leader RNA targets. Moreover, the association of many ANTAR regulators with aspects of nitrogen metabolism provokes intriguing questions about their physiology and evolution.

## Comparison to CAT Domain Antitermination

Proteins of the BglG-LicT-SacY family, present in both gram-negative and gram-positive lineages, control genes for sugar catabolism. These homodimeric proteins contain PRD domains (PTS regulation domain) that place them under control of the phosphotransferase system for sugar uptake [21]. The carboxyl-terminal CAT (co-antiterminator) domain interacts with the RAT (ribonucleic antiterminator) element in the transcribed leader region. The CAT-stabilized antiterminator is mutually exclusive with the terminator structure [22] (Figure 1C and 1D).

The CAT domain is a homodimer of four-stranded β-sheets [23]. Phosphorylation results in massive structural changes [24] that bring the two CAT monomers into proper alignment to interact with a distorted minor grove in the antiterminator hairpin stem [25]. This stem is interrupted by two non-identical asymmetric internal loops, which are recognized similarly by the protein dimer. Sequence analysis suggests that this RNA-binding mode likely is conserved in homologous systems [25].

## How Does ANTAR Bind RNA?

X-ray structures are known for three ANTAR domain proteins, each with a different amino-terminal input domain: AmiR, pseudo-receiver [13]; *Mycobacterium tuberculosis* Rv1626, receiver [26]; and NasR, NIT [27]. Each protein contains an α-helical coiled-coil connecting the two domains, but each structure reveals a different spatial orientation of the ANTAR monomers and a different configuration of the coiled-coil. It is likely that signal is propagated through the coiled-coil to bring the ANTAR monomers into proper alignment for RNA binding [6].

The ANTAR domain adopts a helix-turn-helix conformation, but how it binds RNA is unknown. RNA-binding helix-turn-helix proteins include σ^70^ region 4.2, which binds the −35 region of promoter DNA as well as 6S RNA [28], and Ffh, which binds 4.5 S RNA in the signal recognition particle [29]. Both of these RNA targets form bulged stem-loop structures, so the binding sites have characteristics of dsRNA.

Future progress in understanding ANTAR-mediated antitermination requires knowledge of ANTAR domain conformation and interaction with its RNA target; conformational shifts mediated through the coiled-coil; and kinetics of RNA binding in relation to transcription-driven formation of the P1, P2, and terminator structures.
